# Exopolysaccharide production in *Caulobacter crescentus*: A resource allocation trade-off between protection and proliferation

**DOI:** 10.1371/journal.pone.0190371

**Published:** 2018-01-02

**Authors:** Kathryn L. Herr, Alexis M. Carey, Taylor I. Heckman, Jessenia Laki Chávez, Christina N. Johnson, Emily Harvey, William A. Gamroth, Bridget S. Wulfing, Rachel A. Van Kessel, Melissa E. Marks

**Affiliations:** Department of Biology, Willamette University, Salem, OR, United States of America; University of Massachusetts, UNITED STATES

## Abstract

Complex and interacting selective pressures can produce bacterial communities with a range of phenotypes. One measure of bacterial success is the ability of cells or populations to proliferate while avoiding lytic phage infection. Resistance against bacteriophage infection can occur in the form of a metabolically expensive exopolysaccharide capsule. Here, we show that in *Caulobacter crescentus*, presence of an exopolysaccharide capsule provides measurable protection against infection from a lytic paracrystalline S-layer bacteriophage (CR30), but at a metabolic cost that reduces success in a phage-free environment. Carbon flux through GDP-mannose 4,6 dehydratase in different catabolic and anabolic pathways appears to mediate this trade-off. Together, our data support a model in which diversity in bacterial communities may be maintained through variable selection on phenotypes utilizing the same metabolic pathway.

## Introduction

Exopolysaccharides (EPS) play important roles in the biology of prokaryotes and are associated with increased pathogenicity, stress tolerance, antibiotic resistance, and evasion of predation. These diverse structures share an important common feature: they are composed of sugar monomers that are exported and assembled on the cell surface [1]. The mechanism by which the surface polysaccharides provide protection to cells in natural environments is varied, but can result from greater adhesion, molecular camouflage, or physical barrier formation [2]. However, assembly of extracellular structures is metabolically expensive for cells [1]. The magnitude of the metabolic cost would be expected to dictate the speed at which natural selection could operate and whether species would evolve compensatory phenotypes. Even small differences that only manifest in extreme environments could have notable effects on the phenotypic diversity observed in the nature. In carbon limited, oligotrophic environments, this metabolic expenditure can be particularly disadvantageous [3]. Some bacteria can regulate EPS production in response to changing conditions. In other cases, selection for or against EPS production in different environments may facilitate maintenance of variation in constitutive EPS phenotypes within the population [4–8]. Indeed, a wide variety of EPS phenotypes are found to coexist in complex natural ecosystems.

Lytic bacteriophage pose a strong selective pressure when present in bacterial communities [9]. There is clear evidence that reduced phage susceptibility conferred by the presence of EPS is evolutionarily advantageous in natural environments [3,8,10–12]. So why then do we find naturally occurring strains that do not produce EPS (EPS^-^)? One reason may be the trade-off in metabolic efficiency and the need to prioritize allocation of carbon to cell proliferation rather than to EPS production [13]. Indeed, in many systems EPS^-^ strains do outcompete EPS^+^ strains under planktonic or disturbed growth conditions. However, in the establishment of biofilm communities, EPS^+^ strains are frequently favored [4,14,15]. Thus, in the natural environment, there appear to be competing selective pressures for and against EPS producing strains. These ecologically and evolutionarily relevant differences can appear subtle because the phenotypes are frequently physiological and tightly regulated. Similarly, in the laboratory environment selective pressures for domesticated phenotypes (fast growth, stress tolerance, improved survival) can lead to changes in these same traits in the absence of competing selective forces (i.e. nutrient limitation, infection, or predation) [2,16–20]. In *Caulobacter crescentus* specifically, loss of EPS evolves frequently and can become fixed in the population; the probable mechanism resulting in the evolution of divergent EPS phenotypes between the laboratory strains NA1000 (EPS^+^) and CB15 (EPS^-^) [16,21,22].

In *C*. *crescentus* strain NA1000, EPS is a repeating tetrasaccharide containing mannose, glucose, fucose, and galactose [23]. The presence of EPS gives colonies a smooth, mucoid appearance while strains lacking EPS display a dry, rough colony morphology and are more susceptible to infection by the S-layer phage CR30 [16,24,25]. EPS production in *C*. *crescentus* is regulated during the cell cycle by the second messenger cyclic-di-GMP, but seemingly not in response to environmental conditions [26]. Many of the essential components of the EPS biosynthetic pathway are encoded within a 26kb mobile genetic element (MGE). Deletion of the entire MGE results in strains that are unable to produce EPS (EPS^-^, e.g. NA1000ΔMGE, CB15) [16,24]. Disruption of any single gene encoding a component of the biosynthetic EPS machinery from the chromosome (CCNA_00162, 163, 164, 167, 168, *hvyA*, *pssY*, *pssZ*, or *hfsE*) or the MGE (CCNA_466, 467,469, or 3998) also completely abolishes EPS production [24].

In addition to refining our understanding of the predicted EPS synthesis pathway in *C*. *crescentus*, we report that EPS^+^ strains are more resistant to phage infection at the cost of reduced competitive ability (i.e. slower growth rate) in phage-free laboratory environments. Our results suggest that carbon flux through key metabolic pathways can affect expression of advantageous traits as well as competitive ability under nutrient limitation.

## Results and discussion

### Biosynthesis of fucose is required for EPS synthesis

**Fig 1:**
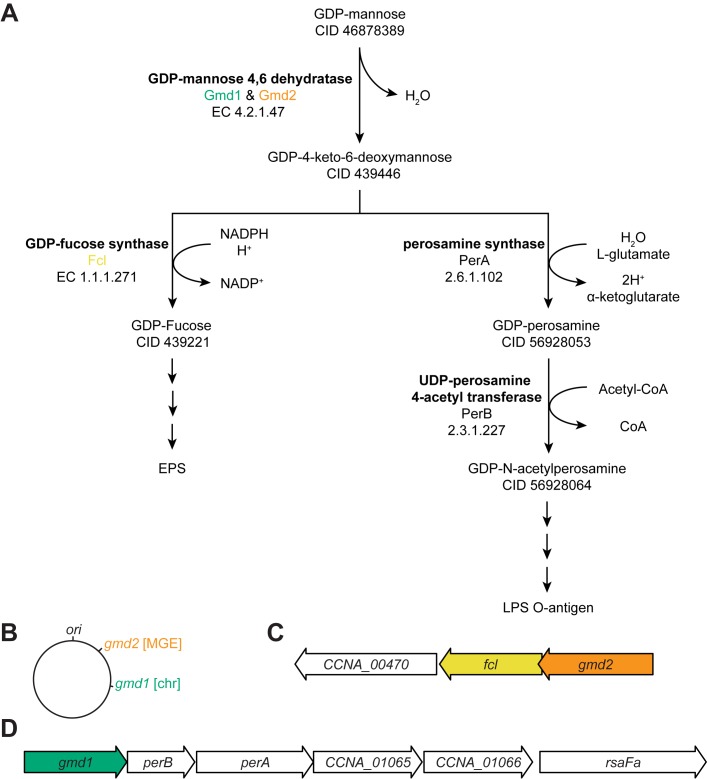
Fucose and N-acetylperosamine monomers required in exopolysaccharide (EPS) and lipopolysaccharide (LPS) assembly in *Caulobacter crescentus* are synthesized by enzymes encoded at two loci. (A) Proposed biosynthetic pathways for synthesis of fucose and N-acetylperosamine monomers. Genomic, enzyme, and pathway data [27,28] support a predicted synthesis pathway for conversion of the GDP-mannose to GDP-fucose and GDP-N-acetylperosamine. Synthesis of both monomers begins with a shared first step in which conversion of GDP-mannose to GDP-4-keto-6-deoxymannose, is catalyzed by Gmd. The GDP-6-keto-6-deoyxmannose intermediate can be converted to GDP-fucose by Fcl or to GDP-N-acetylperosamine by PerA and Per B. Enzyme names are indicated in bold, gene names, and Enzyme Commission (EC) numbers are also indicated. PubChem CID numbers are provided for each predicted precursor/product. (B) The NA1000 genome encodes the enzymes necessary for conversion of GDP-mannose to GDP-fucose or GDP-N-acetylperosamine in operons located at two positions in the genome. The first locus is contained within the mobile genetic element (MGE, orange) and the second on the chromosome (chr, green). The operon structure at each locus is unique, but both operons contain a predicted *gmd* (*gmd2* within the MGE and *gmd1* on the chromosome). (C) The *gmd2* gene (CCNA_00472) contained within the MGE is co-expressed in an operon with *fcl* (CCNA_00471) and an O-antigen polymerase (CCNA_00470) [29], suggesting it is important for the synthesis of GDP-fucose. (D) The *gmd1* (CCNA_01062) encoded on the chromosome is expressed in an operon with *perA* (CCNA_01064) and *perB* (CCNA_01063), as well as two predicted glycosyltransferases (CCNA_01065 and CCNA_1066) and the outer membrane transport protein *rsaFa* [29], consistent with its role in synthesizing GDP-N-acetylperosamine.

Fucose synthesis is required for production of the EPS capsule in *C*. *crescentus* [23]. Biosynthesis of the GDP-fucose monomer requires the activity of GDP-fucose synthase (CCNA_00471, *fcl*), as loss of *fcl* activity also abolishes EPS production (S1 Fig). Using a combination of publicly available data, biosynthetic pathway databases [27,28], and our own genetic analyses we investigated the potential pathway for synthesis of the fucose monomer. We propose that in *C*. *crescentus*, synthesis of the fucose monomer from the GDP-mannose precursor occurs in a two-step process that requires the activities of both of GDP-mannose 4,6 dehydratase (Gmd) and GDP-fucose synthase (CCNA_00471, *fcl*) (Fig 1A). The *C*. *crescentus* genome encodes two predicted *gmd* genes, one within the chromosome (CCNA_01062, *gmd1*, Fig 1B and 1D) and one within the mobile genetic element (MGE; CCNA_00472, *gmd2*, Fig 1B and 1C). This MGE also contains many of the genes necessary for EPS assembly and export (CCNA_00466, 467,469, and 3998) (S1 Fig). Both *gmd* genes are expressed within operons [29] encoding precursor synthases and glycosyltransferases predicted to be important in polysaccharide synthesis and assembly processes [24] (Fig 1B and 1C).

**Fig 2:**
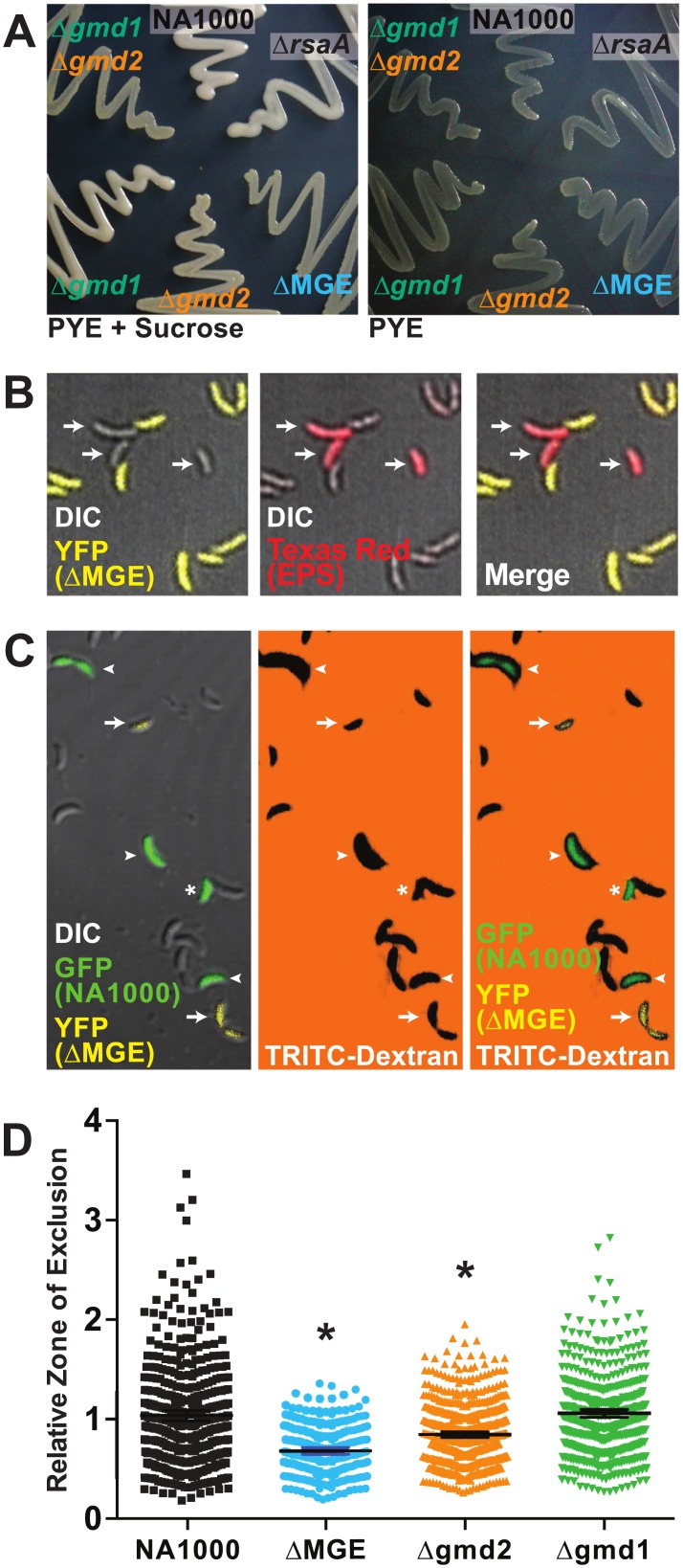
GDP-mannose 4,6 dehydratase (Gmd) activity is required for EPS synthesis in *C*. *crescentus*. (A) EPS phenotypes on PYE-sucrose (left) and PYE media (right). NA1000 has a fully mucoid (EPS^+^) phenotype and NA1000ΔMGE has a dry, non-mucoid (EPS^-^) phenotype when cultured on PYE-sucrose (left). The NA1000Δ*gmd1* and NA1000Δ*gmd2* single mutants show slightly reduced or markedly reduced EPS expression, respectively. The NA1000Δ*gmd2*Δ*gmd1* double mutant has a dry, non-mucoid (EPS^-^) phenotype, grows slowly, and shows slightly reduced colony size. The NA1000Δ*rsaA* mutant has a wildtype, fully mucoid (EPS^+^) phenotype. The different EPS phenotypes are subtle and do not photograph well when they are grown on plain PYE media (right). (B) Merged Differential Interference Contrast (DIC) and fluorescence images of mixed EPS^+^ (NA1000, non-fluorescent) and EPS^-^ (NA1000ΔMGE, YFP-expressing, yellow) cells stained with a Texas Red conjugated lectin that specifically binds fucose residues (*Lotus tetragonolobus*, red). The lectin binds to EPS^+^ cells, but not EPS^-^ cells. (C) Representative DIC and fluorescence images of mixed EPS^+^ (NA1000, GFP-expressing, green) and EPS^-^ (NA1000ΔMGE, YFP-expressing, yellow) showing TRITC-Dextran exclusion. The black regions surrounding the cells correspond with the capsule (EPS) and EPS^+^ (GFP^+^ cells, indicated with arrowheads) cells show larger zones of exclusion than EPS^-^ (YFP^+^ cells, indicated with arrows) cells in this negative stain assay. Only isolated cells unambiguously expressing a fluorescent protein (arrows, arrowheads) were included in quantitative analyses while non-fluorescent and adjacent cells (asterisk) were excluded. (D) Quantitative analysis of the zones of exclusion from the experiment described in panel (C) show that NA1000 (black squares) and NA1000Δ*gmd1* (green triangles) make more EPS than NA1000ΔMGE (blue circles) and NA1000Δ*gmd2* (yellow triangles) (ANOVA F(3, 1657) = 63.19, P < 0.0001, NA1000 vs. NA1000ΔMGE t(1675) = 10.85 p < 0.0001, NA1000 vs. NA1000Δ*gmd2* t(1657) = 7.241 p < 0.0001, NA1000 vs. NA1000Δ*gmd1* t(1657) = 0.8581 p > 0.9999) when grown under the same conditions.

The fucose monomer (GDP-fucose) is synthesized in a two-step process from a mannose precursor (GDP-mannose). The first step in the synthesis of fucose is likely catalyzed by Gmd1/Gmd2 and yields the intermediate GDP-4-keto-6-deoxymannose (Fig 1A). *gmd2* is expressed in an operon with *fcl* and another predicted glycosyltransferase (CCNA_00470) [24,29], suggesting that these proteins are functionally related (Fig 1C). Without functional Fcl (NA1000Δ*fcl*), *Caulobacter* cells are unable to synthesize EPS (S1 Fig). The GDP-4-keto-6-deoxymannose intermediate also appears to be the precursor for synthesis of GDP-N-acetylperosamine, a major component of the *C*. *crescentus* lipopolysaccharide O-antigen [30], through the activity of two adjacent genes (CCNA_01063, *perB* and CCNA_01064, *perA*). The *gmd1* operon structure in which *gmd1*, *perA*, and *perB*, are co-expressed in an operon with two predicted glycosyltransferases involved in lipopolysaccharide O-antigen (CCNA_01065 and CCNA_01066) as well as the outer membrane transport protein *rsaFa* [29] lends support to the idea that GDP-mannose is the precursor for synthesis of GDP-N-acetylperosamine (Fig 1D). The predicted functions of Gmd1 and Gmd2 are further supported by the observation that mutants entirely lacking Gmd activity (NA1000Δ*gmd*1Δ*gmd2*) display EPS^-^ and S-layer shedding phenotypes consistent with disrupted EPS and O-antigen polysaccharide synthesis (Fig 1A, Fig 2A, and Table 1). In the absence of the O-antigen, the *Caulobacter* S-layer protein (encoded by *rsaA*) cannot assemble on cellular surfaces and is shed into the media where it visibly aggregates on the test tube walls [31,32]. In addition, the mucoid phenotype of NA1000Δ*rsaA* [33] confirms that that proper assembly of the S-layer is not required for EPS synthesis or assembly (Fig 2A).

**Table 1:** Strains and plasmids.

Strain or plasmid	Description and relevant characteristics	Source
***Caulobacter strains***		
NA1000	NA1000; wildtype EPS^+^ strain	L. Shapiro [16,22]
NA1000ΔMGE	NA1000 with a deletion of the MGE (EPS^-^)	[16]
NA1000Δ*fcl*	NA1000 with an in-frame deletion of *fcl* (CCNA_00471)	This study
NA1000Δ*469*	NA1000 with an in-frame deletion of CCNA_00469	This study
NA1000Δ*gmd2*	NA1000 with an in-frame deletion of *gmd2* (CCNA_00472)	This study
NA1000Δ*gmd1*	NA1000 with an in-frame deletion of *gmd1* (CCNA_01062)	This study
NA1000Δ*gmd1*Δ*gmd2*	NNA1000 with in-frame deletions of *gmd1* and *gmd2* (CCNA_01062, CCNA_00472)	This study
NA1000Δ*xylB*	NA1000 with an in-frame deletion of *xylB;* cannot metabolize xylose	This study
NA1000ΔMGEΔxylB	NA1000ΔMGE with an in-frame deletion of *xylB*, cannot metabolize xylose	This study
NA1000Δ*gmd2*Δ*xylB*	NA1000Δ*gmd2* with an in-frame deletion of *xylB*,; cannot metabolize xylose	This study
NA1000Δ*gmd1*Δ*xylB*	NA1000Δ*gmd1* with an in-frame deletion of *xylB*; cannot metabolize xylose	This study
NA1000Δ*xylB* XylX: : pXCFPC-2	NA1000Δ*xylB* expressing CFP under the control of P_xyl_	This study
NA1000Δ*xylB* XylX: : pXGFPC-2	NA1000Δ*xylB* expressing GFP under the control of P_xyl_	This study
NA1000ΔMGEΔ*xylB* XylX: : pXCHYC-2	NA1000Δ*xylB* expressing mCherry under the control of P_xyl_	This study
NA1000ΔMGEΔ*xylB* XylX: : pXYFPC-2	NA1000ΔMGEΔ*xylB* expressing YFP under the control of P_xyl_	This study
NA1000ΔMGEΔ*xylB* XylX: : pXCFPC-2	NA1000ΔMGEΔ*xylB* expressing CFP under the control of P_xyl_	This study
NA1000ΔMGEΔ*xylB* XylX: : pXGFPC-2	NA1000ΔMGEΔ*xylB* expressing GFP under the control of P_xyl_	This study
NA1000Δ*gmd2*Δ*xylB* XylX: : pXCHYC-2	NNA1000Δ*gmd2*Δ*xylB* expressing mCherry under the control of P_xyl_	This study
NA1000Δ*gmd2*Δ*xylB* XylX: : pXYFPC-2	NA1000Δ*gmd2*Δ*xylB* expressing YFP under the control of P_xyl_	This study
NA1000Δ*gmd2*Δ*xylB* XylX: : pXCFPC-2	NA1000Δ*gmd2*Δ*xylB* expressing CFP under the control of P_xyl_	This study
NA1000Δ*gmd2*Δ*xylB* XylX: : pXGFPC-2	NA1000Δ*gmd2*Δ*xylB* expressing GFP under the control of P_xyl_	This study
NA1000Δ*gmd2*Δ*xylB* XylX: : pXCHYC-2	NA1000Δ*gmd2*Δ*xylB* expressing mCherry under the control of P_xyl_	This study
NA1000Δ*gmd2*Δ*xylB* XylX: : pXYFPC-2	NNA1000Δ*gmd2*Δ*xylB* expressing YFP under the control of P_xyl_	This study
NA1000Δ*gmd1*Δ*xylB* XylX: : pXCFPC-2	NA1000Δ*gmd1*Δ*xylB* expressing CFP under the control of P_xyl_	This study
NA1000Δ*gmd1*Δ*xylB* XylX: : pXGFPC-2	NA1000Δ*gmd1*Δ*xylB* expressing GFP under the control of P_xyl_	This study
NA1000Δ*gmd1*Δ*xylB* XylX: : pXCHYC-2	NA1000Δ*gmd1*Δ*xylB* expressing mCherry under the control of P_xyl_	This study
NA1000Δ*gmd1*Δ*xylB* XylX: : pXYFPC-2	NNA1000Δ*gmd1*Δ*xylB* expressing YFP under the control of P_xyl_	This study
NA1000Δ*RsaA*	NA1000 with an in-frame deletion of *rsaA*; does not secrete RsaA or assemble an S-Layer	J. Smit [33]
**Plasmids**		
pNPTS138	Allele replacement vector	M.R.K. Alley [34]
pNPTS138-Δ*xylB*	Allele replacement vector carrying an in-frame deletion construct for *xylB*	This study
pNPTS138-Δ*fcl*	Allele replacement vector carrying an in-frame deletion construct for *fcl* (CCNA_00471)	This study
pNPTS138-Δ*469*	Allele replacement vector carrying an in-frame deletion construct for CCNA_00469	This study
pNPTS138-Δ*gmd1*	Allele replacement vector carrying an in-frame deletion construct for *gmd1* (CCNA_01062)	This study
pNPTS138-Δ*gmd2*	Allele replacement vector carrying an in-frame deletion construct for *gmd2* (CCNA_00472)	This study
pXCFPC-2	For generating xylose-inducible CFP expression or C-terminal CFP fusion proteins encoded at the *xylX* locus; Kan^R^	M. Thanbichler [35]
pXGFPC-2	For generating xylose-inducible GFP expression or C-terminal GFP fusion proteins encoded at the *xylX* locus; Kan^R^	M. Thanbichler [35]
pXCHYC-2	For generating xylose-inducible mcherry expression or C-terminal mCherry fusion proteins encoded at the *xylX* locus; Kan^R^	M. Thanbichler [35]
pXYFPC-2	For generating xylose-inducible YFP expression or C-terminal YFP fusion proteins encoded at the *xylX* locus; Kan^R^	M. Thanbichler [35]

Our analyses also suggest that *C*. *crescentus*’ ability to produce EPS is dependent on the nutrient environment and can be fine-tuned by altering the carbon flux through the Gmd1/Gmd2 pathway. Colonies grown on media supplemented with sugar (sucrose, glucose) are visibly more mucoid than those cultured on media containing only peptone and yeast extract (Fig 2A and Table 1). In strains lacking the MGE (NA1000ΔMGE), EPS cannot be detected using fluorescently conjugated lectins (*Lotus tetragonolobus*) that specifically recognize the fucose component of the EPS (Fig 2B). Furthermore, in the absence of either Gmd1 (NA1000Δ*gmd1*) or Gmd2 (NA1000Δ*gmd2*) activity, *C*. *crescentus* cells appear to produce less EPS, but colonies still appear mucoid (Fig 2A). The NA1000Δ*gmd1* strain is more mucoid than the NA1000Δ*gmd2* strain, implying that Gmd2 may have higher activity.

We visualized and measured zones of dextran exclusion [24,36] to further examine the quantitaive effect of different levels of Gmd activity on EPS production. Using this approach, we find that NA1000 (EPS^+^) cells produce zones of exclusion that are significantly larger than those of EPS^-^ NA1000ΔMGE and NA1000Δ*gmd2* cells (ANOVA F(3, 1657) = 63.19, NA1000 vs. NA1000ΔMGE, t(1657) = 10.85, p < 0.001, NA1000 vs. NA1000Δ*gmd2* t(1657) = 7.241, p< 0.001, Fig 2C and 2D). Full producer NA1000 cells were not significantly different than the higher-producing intermediate NA1000Δ*gmd1* (NA1000 vs. NA1000Δ*gmd1*, t(1657) = 0.8581, p > 0.9999, Fig 2D). These results are consistent with the qualitative observations of mucoidy on high-sugar PYE plates (Fig 2A). Because we are unable to quantitatively distinguish between the EPS phenotypes of NA1000 and NA1000Δ*gmd1*, this mutant was not included in subsequent analyses.

### EPS-producing cells are less susceptible to bacteriophage infection

**Fig 3:**
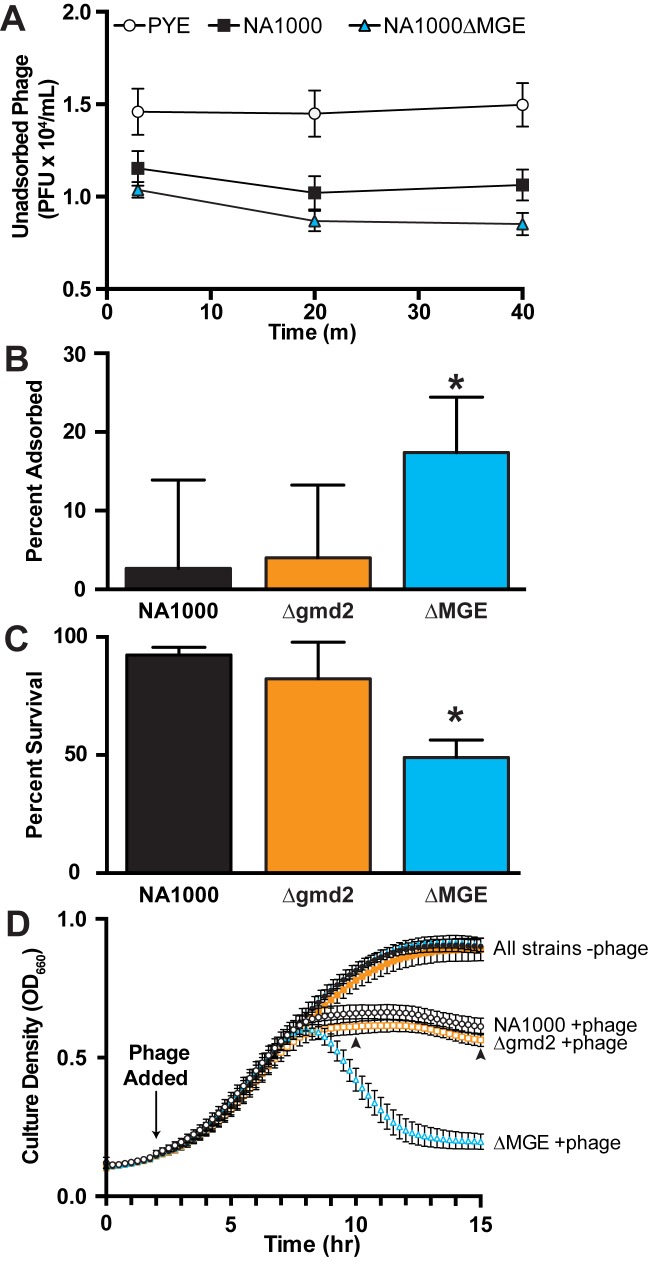
*C*. *crescentus* EPS inhibits binding and provides partial resistance to bacteriophage CR30. (A) Cultures of NA1000ΔMGE adsorb more phage CR30 than NA1000. In a timecourse experiment where phage CR30 was added to *C*. *crescentus* cultures, sampled immediately (60–90 seconds), at 20 minutes, and at 40 minutes. Unadsorbed phage were quantified. Both samples containing *C*.*crescentus* cells adsorbed phage quickly and show significantly fewer remaining plaque forming units in the lysate (NA1000, black squares and NA1000ΔMGE, blue triangles) relative to the control (PYE, open circle). Throughout the experiment, EPS^-^ (NA1000ΔMGE) cells continue to adsorb more phage than EPS^+^ (NA1000) cells. (B) The presence of EPS on cellular surfaces reduces phage CR30 adsorption. Adsorption experiments were carried out as in (A) and percent adsorption was measured as the concentration of viable plaque forming units present in the sample at 40 minutes relative to the number present at the initial sampling point. EPS^+^ (NA1000, black bar) and intermediate EPS (NA1000Δ*gmd2*, orange bar) cells adsorb fewer phage after 40 minutes, than EPS^-^ (NA1000ΔMGE blue bar) cells (ANOVA F(2, 42) = 3.506, P = 0.0391, NA1000 vs. NA1000ΔMGE t(42) = 2.395, p = 0.0423, NA1000 vs. NA1000Δ*gmd2* t(42) = 0.2207, p > 0.9999). (C) Survival is lower in strains that adsorb more phage. Cultures of EPS^+^, intermediate, or EPS^-^ cells (NA1000, NA1000Δ*gmd2*, or NA1000ΔMGE, respectively) were challenged with phage CR30, and incubated for 18 hours. Cell density (OD_660_) was compared with no phage controls (ANOVA F(2, 61) = 45.79, p < 0.0001, NA1000 vs. NA1000ΔMGE t(61) = 9.288, p < 0.0001, NA1000 vs. NA1000Δgmd2 F(2, 61) = 1.708, p = 0.1855). (D) The dynamics of population response phage to CR30 infection are influenced by EPS phenotype. As expected, all strains tested showed some susceptibility to phage CR30 and do not grow to the same density as phage-free cultures. As seen in previous experiments, (A-C), the presence of EPS is largely protective against phage CR30 infection. With larger sample sizes in this high throughput assay, we measured a small increase in phage CR30 susceptibility in NA1000Δ*gmd2* relative to NA1000 (at 10 hours: t(66) 2.357, p = 0.0214; at 15 hours: t(66) = 2.582, p = 0.0121, indicated with arrowheads).

**Fig 4:**
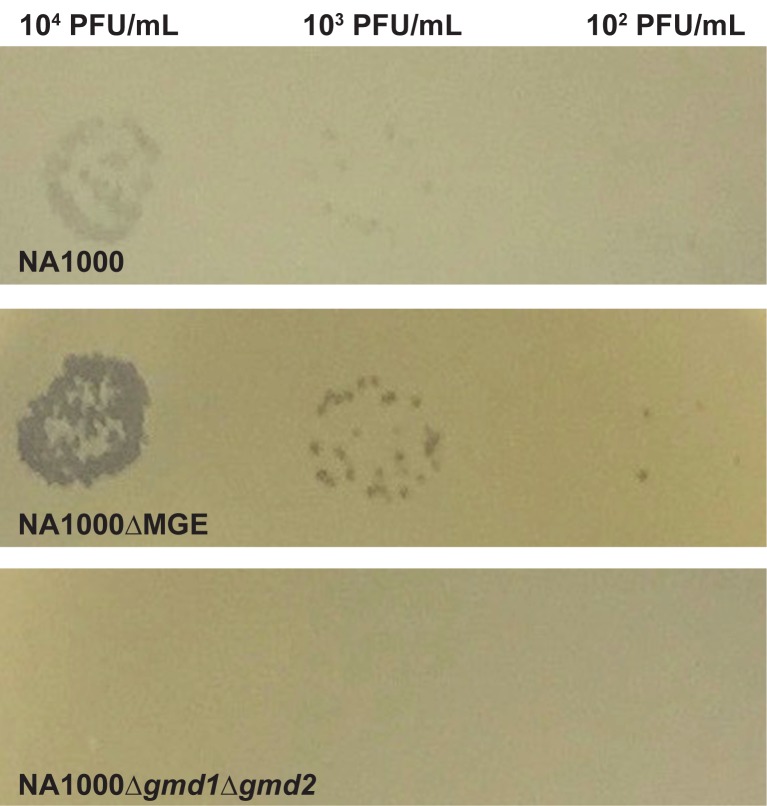
Gmd activity is required for maintenance of S-layer dependent phage CR30 susceptibility. Both NA1000 (EPS^+^, top) and NA1000ΔMGE (EPS^-^, middle) support plaque formation when challenged with phage CR30 lysates (10^2^–10^4^ pfu/mL). Note that NA1000ΔMGE is about 10-fold more sensitive to CR30 infection under these conditions, consistent with previous findings [16,24]. In contrast, NA1000Δ*gmd*1Δ*gmd2* (EPS^-^, bottom) does not support phage CR30 plaque formation; a result that is consistent with the hypothesis that this strain sheds the S-layer because it is unable to synthesize N-acetylperosamine and assemble normal LPS O-antigen (Fig 1) [30,31].

Cells that produce EPS could be less susceptible to infection with bacteriophage CR30 (Fig 3) because the EPS may physically obscure the S-layer binding sites used by CR30 [37]. To test this hypothesis, we measured phage adsorption to the surface of EPS^+^ and EPS^-^ strains of *C*. *crescentus*. The EPS^+^ NA1000 strain adsorbs less phage than the EPS^-^ NA1000ΔMGE strain (Fig 3A). Note that the phage binding kinetics are rapid and most adsorption occurs between the addition of the phage and the first sample collection (at 60–90 seconds). The total number of unadsorbed phage recovered from the no cell control sample (PYE) is significantly (approximately 30%) higher than that recovered from either NA1000 or NA1000ΔMGE samples (ANOVA F(2, 41) = 25.77, PYE vs. NA1000 t(41) = 4.956, p < 0.0001, PYE vs. NA1000ΔMGE t(41) = 6.958, p < 0.0001). This result indicates that the phage begin to adsorb to cells very quickly. Over the next 40 minutes in this experiment, phage continue to adsorb more effectively to NA1000ΔMGE (EPS^-^) than NA1000 (EPS^+^). In addition, EPS^+^ NA1000 and intermediate NA1000Δ*gmd2* cells adsorb significantly fewer CR30 virions than EPS^-^ NA1000ΔMGE cells within a 40 minute incubation period (Fig 3B, ANOVA F(2, 42) = 3.506, P = 0.0391, NA1000 vs. NA1000ΔMGE t(42) = 2.395, P = 0.0423, NA1000 vs. NA1000Δ*gmd2* t(42) = 0.2207, P > 0.9999). Phage CR30 infection is also more successful and results in higher lethality in NA1000ΔMGE (EPS^-^) than in NA1000 (EPS^+^) or NA1000Δ*gmd2* (Fig 3C, ANOVA F(2, 61) = 45.79, P < 0.0001, NA1000Δ vs. NA1000ΔMGE t(61) = 9.288, p < 0.0001; NA1000 vs. NA1000Δ*gmd2* t(61) = 1.708, p = 0.1855). Despite their EPS^-^ phenotype, NA1000Δ*gmd1*Δ*gmd2* cells are almost completely resistant to phage CR30 infection. No lysis is observed in lawns of NA1000Δ*gmd1*Δ*gmd2* when challenged with phage lysates at concentrations that generate significant clearing in both NA1000 and NA1000ΔMGE (Fig 4). These data corroborate the hypothesis that this mutant exhibits an S-layer shedding phenotype, resulting from the inability to synthesize the LPS O-antigen required for proper S-layer assembly [31,32]. Together our results are consistent with the hypothesis that the S-layer in the EPS^+^ strains is physically obscured by the presence of EPS.

In examining the adsorption and endpoint survival data (Fig 2B and 2C, respectively), an intriguing trend emerged: NA1000Δ*gmd2* (intermediate EPS) shows slightly increased adsorption and reduced survival relative to the full producing EPS^+^ NA1000 strain. To further assess the role of EPS in phage susceptibility, we followed bacterial growth and lysis over a 15-hour period in cultures challenged with phage CR30. We tested three strains with varying levels of EPS: NA1000 (EPS^+^), NA1000ΔMGE (EPS^-^), and NA1000Δ*gmd2* (intermediate EPS). As expected, final culture ODs were significantly lower than the paired, no phage control cultures. The EPS^-^ strain shows rapid lysis starting approximately five to six hours after phage addition. In contrast, the EPS^+^ and intermediate strains do not show the rapid lysis, but appear to reach a steady state around the same time point. While both EPS producing strains exhibit similar dynamics, there is a small, but consistent difference in overall culture density that mirrors the qualitative and quantitative difference in EPS phenotypes. At both 10 and 15 hours (8 and 13 hours after phage CR30 addition), more NA1000 than NA1000Δ*gmd2* cells remain intact (at 10 hours: t(66) 2.357, p = 0.0214; at 15 hours: t(66) = 2.582, p = 0.0121, Fig 3D). If NA1000Δ*gmd2* cells also produce less O-antigen as a result of reduced Gmd activity, it is possible they could anchor less S-layer rendering them more resistant to phage CR30 than NA1000. However, our results indicate that NA1000Δ*gmd2* is slightly more susceptible to phage CR30 than NA1000. If NA1000Δ*gmd2* were less susceptible to phage because of reduced O-antigen and also more susceptible because of reduced EPS, then a strain producing wildtype amounts of O-antigen, but reduced EPS would be more susceptible than our NA1000Δ*gmd2*. Therefore, a reduction in O-antigen anchored S-Layer could potentially mask some of EPS effect. Together our results suggest that that production of some EPS is almost as effective at protecting against phage CR30 infection as full, wildtype production in the nutrient replete, laboratory environment.

### EPS-producing cells are less competitive in the absence of phage

If EPS production provides such a dramatic and clear survival advantage, why is it that some non-mucoid EPS^-^ strains survive in nature? To investigate this question, we measured growth rates of our EPS^-^ and EPS^+^ strains as a proxy for the metabolic cost of EPS production. Because *C*. *crescentus* is an oligotrophic species naturally residing in low nutrient environments, limitation of carbon sources should be a major regulator of cellular growth rate [38]. In an EPS^+^ strain, some of those limited carbon resources are modified and exported to the cell surface where they are unavailable for use in cellular maintenance or growth. We measured growth rates of EPS^+^ (NA1000) and EPS^-^ (NA1000ΔMGE) in regular rich media (PYE) and found a measurable, but not statistically significant difference in the average doubling times for these strains (NA1000 145.4 ± 1.311 minutes (n = 49), ΔMGE 142.9 ± 1.396 minutes (n = 49); t(96) = 1.272, p = 0.206). This small, yet reproducible difference in growth rates could have competitive advantages for EPS^-^ strains or species growing under nutrient-limited conditions.

To measure the benefit of a 1.7% (2.5 minute per generation) decrease in doubling time, we directly competed NA1000, NA1000ΔMGE, and NA1000Δ*gmd2* strains (Table 1) in pairwise combinations. In these experiments, strains with GFP or mCherry under the control of the xylose-inducible promoter P_xyl_ [35] were mixed 1:1 (GFP strain: mCherry strain) and co-cultured. Each day, the mCherry and GFP fluorescence intensities were measured and mixed cultures were diluted with fresh media and cultured again. This process was repeated daily for the duration of the experiment. To achieve consistent fluorescent protein expression in rich meida, we generated Δ*xylB* mutants in which the first gene in the xylose degradation pathway is non-functional, disabling xylose metabolism (Table 1) [39,40]. Thus, the xylose inducer remains intact and able to activate the xylX promoter throughout the experiment, even at low xylose concentrations (0.01–0.1%). There is some variability in fluorescence between these strains, but each strain has a consistent and reproducible brightness independent of the strain background or EPS phenotype (S2 Fig).

**Fig 5:**
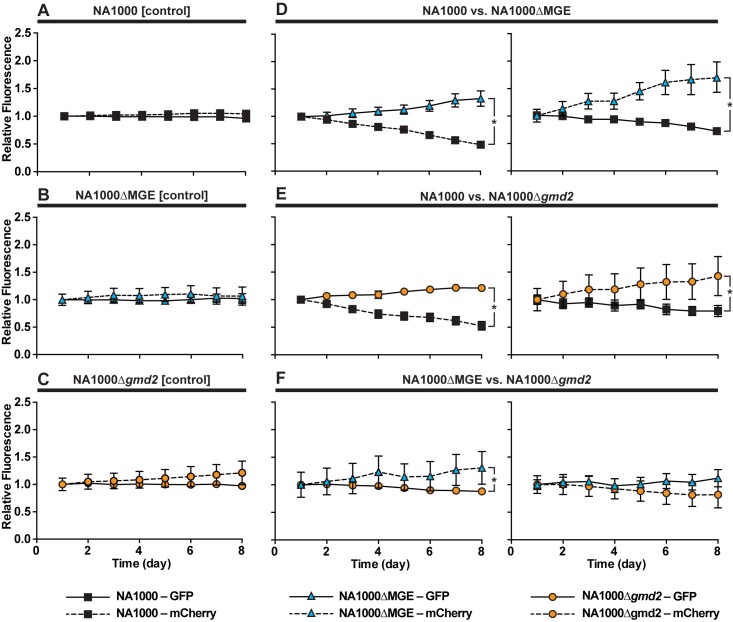
EPS production may be disadvantageous in phage-free environments. Strains labeled with two different fluorophores (GFP, mCherry) were mixed and relative fluorescence measured over the course of 8 days. (A–C) In control experiments where strains of the same genetic background expressing different fluorescent proteins were mixed, no significant change in the ratio of GFP:mCherry fluorescence was detected. Conversely, when strains of different genotypes were mixed, those producing more EPS appear to be at a competitive disadvantage (D) NA1000 and NA1000ΔMGE, (E) NA1000 and NA1000Δ*gmd2*, or (F) NA1000ΔMGE and NA1000Δ*gmd2*. The difference in fluorescence ratio between GFP and mCherry was assessed at the beginning (Day 1) and end (Day 8) of the experiment using an ANOVA with Bonferroni correction for multiple comparisons. Comparisons that resulted in significant differences are indicated with an asterisk (*) in the figure. (Day 1: ANOVA F(17, 558) = 0.0009178, P > 0.9999; Day 8: ANOVA F(17, 554) = 16.40, P < 0.0001, NA1000-GFP vs. NA1000-mCherry, t(554) = 1.051, p > 0.9999, NA1000ΔMGE-GFP vs. NA1000ΔMGE-mCherry t(554) = 0.5479, p > 0.9999, or NA1000Δ*gmd2*-GFP vs. NA1000Δ*gmd2*-mCherry t(554) = 2.479, p = 0.1213, NA1000-mCherry vs. NA1000ΔMGE-GFP t(554) = 8.892, p = < 0.0001, NA1000-GFP vs. NA1000ΔMGE-mCherry t(554) = 10.11, p = < 0.0001, NA1000-mCherry vs. NA1000Δ*gmd2*-GFP t(554) = 5.038, p = < 0.0001, NA1000-GFP vs. NA1000Δ*gmd2*-mCherry t(554) = 4.655, p = < 0.0001, NA1000ΔMGE-mCherry vs. NA1000Δ*gmd2*-GFP t(554) = 3.099, p = < 0.0184, NA1000ΔMGE-GFP vs. NA1000Δ*gmd2*-mCherry t(554) = 2.201, p = 0.2532).

At the start of the experiment (Day 1) there are no differences in the ratio of GFP to mCherry signals in any of the experimental configurations (Fig 5, ANOVA F(17, 558) = 0.0009178, P > 0.9999). No change in the ratio of GFP:mCherry signals was detected in control experiments by the completion of the experiment (Day 8), suggesting that neither fluorescent marker has a significantly different metabolic cost (Fig 5A–5C (Day 8: ANOVA F(17, 554) = 16.40, P < 0.0001, NA1000-GFP vs. NA1000-mCherry, t(554) = 1.051, p > 0.9999, NA1000ΔMGE-GFP vs. NA1000ΔMGE-mCherry t(554) = 0.5479, p > 0.9999, or NA1000Δ*gmd2*-GFP vs. NA1000Δ*gmd2*-mCherry t(554) = 2.479, p = 0.1213). In contrast, when strains with different EPS phenotypes were competed (NA1000 vs. NA1000ΔMGE, NA1000 vs. NA1000Δ*gmd2*, or NA1000ΔMGE vs. NA1000Δ*gmd2*) the fluorescent signal of the strain producing more EPS decreases relative to the signal for the competing strain (Fig 5D–5F, ANOVA F(17, 554) = 16.4, NA1000-mCherry vs. NA1000ΔMGE-GFP t(554) = 8.892, p = < 0.0001, NA1000-GFP vs. NA1000ΔMGE-mCherry t(554) = 10.11, p = < 0.0001, NA1000-mCherry vs. NA1000Δ*gmd2*-GFP t(554) = 5.038, p = < 0.0001, NA1000-GFP vs. NA1000Δ*gmd2*-mCherry t(554) = 4.655, p = < 0.0001, NA1000ΔMGE-mCherry vs. NA1000Δ*gmd2*-GFP t(554) = 3.099, p = < 0.0184, NA1000ΔMGE-GFP vs. NA1000Δ*gmd2*-mCherry t(554) = 2.201, p = 0.2532). The difference in fluorescent protein signal continues to diverge over the course of the multi-day experiment. In one of the 285 mixed cultures in this set of experiments, one of the two strains (NA1000ΔMGE) may have acquired an advantageous mutation and rapidly increases in frequency relative to the competitor (NA1000) and appears to reach fixation by day 7 (S3 Fig). In none of the remaining 284 trials is a similar pattern observed (S3 Fig), suggesting that the change in strain frequency in these experiments is almost exclusively a result of selection on the existing variation and the competitive disadvantage of EPS. The competitive advantage of NA1000ΔMGE or NA1000Δ*gmd2* relative to NA1000 is considerable, while the subtle advantage of NA1000ΔMGE relative to NA1000Δ*gmd2* was statistically significant in only one of the two experimental configurations (NA1000ΔMGE-GFP vs. NA1000Δ*gmd2*-mCherry, Fig 5F). This result suggests that the competitive cost of EPS synthesis depends on the amount of EPS produced. These data are consistent with the hypothesis that synthesis of EPS under these growth conditions is metabolically costly. It is also possible that the absence of EPS increases the small molecule permeability of the cellular surface resulting in increased nutrient availability in these cells. Furthermore, the resulting 1.7% (2.5 minute per generation) difference in doubling times between EPS^+^ and EPS^-^ cells can significantly alter allele frequencies in populations experiencing competitive growth conditions in the absence of bacteriophage or other EPS-favoring factors.

## Conclusions

Carbon flux through key metabolic pathways can affect competitive ability in environments with different selective regimes. In *Caulobacter crescentus*, a mobile genetic element encodes many components of a biosynthetic pathway for exopolysaccharide production and assembly. EPS confers resistance to some types of bacteriophage (i.e. CR30) because it obscures the paracrystalline S-layer binding sites on the cell surface. When carbon resources are abundant (i.e. media supplemented with sugar), the capable cells produce more EPS. This phenotype may result from the altered carbon flux through the necessary downstream anabolic pathways, including GDP-mannose 4,6 dehydratase. Alternatively, when carbon resources are scarce (i.e. plain media) it is likely that a higher proportion of the carbon is needed in catabolic pathways required for cell maintenance and survival, a condition that would be exacerbated in the natural oligotrophic environment. However, allocating carbon to cell growth and division processes could be maladaptive when bacteriophage in the environment are abundant and protection from infection becomes necessary [10,41]. When these competing pressures are predictable or context-dependent, species may evolve the ability to regulate EPS expression. In other cases, variable selection pressures may lead to heterogeneity and diversity within the population, perhaps mediated by horizontal gene transfer or loss of mobile genetic elements [8,42,43]. The domestication history of the common laboratory strains of *C*. *crescentus* (NA1000 and CB15) is consistent with the hypothesis that this trade-off is the reason EPS^-^ phenotypes evolve frequently in laboratory strains [16]. Specifically, the spontaneous loss of the MGE results in cells with an advantage, as they no longer bear the metabolic cost of EPS production. Consequently, within a few generations of laboratory culture these EPS^-^ cells can outcompete, or at least co-exist with the original EPS^+^ ancestor. In addition, within the *Caulobacter* group there is variation in the EPS phenotypes of natural isolates suggesting that this trade-off and spectrum of selective pressures may be a significant contributor to the maintenance of diversity in bacterial populations.

## Materials and methods

### Strains, media, and culture conditions

Strains and plasmids are listed in Table 1. Rich medium was PYE (0.2% Bacto peptone, 0.1% yeast extract, 1 mM MgSO_4_, 0.5 mM CaCl_2_). Solid medium was PYE with 1.5% Bacto agar [44]. Top agar was PYE with 0.3% Bacto agar and 0.3% glucose. Minimal medium was M2G or M2X (20 mM Na_2_HPO_4_, 20 mM KH_2_PO_4_, 9.3 mM NH_4_Cl, 0.5 mM MgSO_4_, 0.5 mM CaCl_2_, 1 μM FeSO_4_, 1 μM EDTA, 0.2% glucose or 0.2% xylose). Endpoint phage survival experiments (Fig 3C) were conducted in M_15_HIGG medium (0.31% glucose, 0.09% L-glutamate (monosodium salt), 1.25 mM phosphate, 3.1 mM imidazole, 0.025% ammonium chloride, 0.5% Hunter’s mineral salts) [45]. High-sugar PYE medium was PYE with 1.5% glucose or 3% sucrose. All cultures were grown at 30°C, unless otherwise noted.

### Strain construction

Allele substitution (deletion) strains were constructed using a standard double-crossover allele replacement strategy. The Δ*gmd1* and Δ*gmd2* alleles were generated using a PCR-based approach. Briefly, flanking regions (500 bp) up and downstream of each target gene were amplified and joined using overlap-extension PCR with restriction sites (UNDERLINE), overhangs (lower case), and overlaps (**BOLD**) engineered into the primers (*gmd1*_upF: tctACTAGTGTGATCCAGGCGCATTTC, *gmd1*_upR: **CGCTCATCA****GATATC**TTTCGCCATGCGTATCC, *gmd1*_dnF: **ATGGCGAAA****GATATC**TGATGAGCGCTTCCCTC, *gmd1*_dnR: tataGAATTCATCGTGACGCCAGGTATGAC, *gmd2*_upF: tataACTAGTCCGATGCCATAGAGATTGGT, *gmd2*_upR: **TGC****GGTACCTGC**AATGCCGAATGAcgtgatct, *gmd2*_dnF: **GCA****GGTACC****GCA**TTCAACCGTCATTAGGTCCC, *gmd2*_dnR: tataACTAGTATAATGATGGGGTGGCTGAG, CCNA_00469_upF: tataACTAGTGAGCCGTGCGTGATATCTG, CCNA_00469_upR: **TGC****GGTACC****TGC**CTTCACGCCTAGtcaggtga, CCNA_00469_dnF: **GCAGGTACCGCA**ACAATCGCTCAAaaatgggg, CCNA_00469_dnR: tataACTAGTGCCGTCGCTATGAATATTGTG, *fcl*_upF: tataACTAGTGTCGTACGCCGTAAACTGGT, *fcl*_upR: **TGC****GGTACC****TGC**TCGGCCCAATAAaaactagc, *fcl*_dnF: **GCA****GGTACC****GCA**CCCGGGCGACATtgatcatg: *fcl*_dnR: tataACTAGTTCCTGTTCAATCACGAGAGC). To build the Δ*xylB* allele, flanking regions (500bp) up and downstream of each target gene were identified and a synthetic, in-frame, mutant allele was designed and synthesized as a gBlocks gene fragment (IDT, Coralville, IA) with restriction sites engineered into the 5’ and 3’ ends (sequence available upon request). The PCR product or gBlock was digested with the appropriate restriction enzymes (NEB, Ipswich, MA), cloned into the suicide knockout plasmid pNTPS138 [34], and transferred to the appropriate destination strain using a triparental mating strategy. Counterselection for the second chromosomal crossover event resulting in allele replacement was selected by plating cells that had been outgrown overnight in nonselective liquid medium on PYE-sucrose plates. PCR amplification and direct sequencing of the product verified allele substitutions. PCR was carried out using Q5 Hot Start High-Fidelity 2X Master Mix (NEB, Ipswich, MA) with 0.5 to 1.0 μM primer and the template (0.5 to 1.0 μl fresh *Caulobacter* culture). We used the following thermal cycling protocol: 98°C for 30 s; 30 cycles of 98°C for 10 s, 55°C for 20 s, and 72°C for 30 s/kb; 72°C for 5 m; and maintenance at 4°C. When used as a template for Sanger sequencing, 10 μl of the fresh PCR product was mixed with 8.5 μL nuclease free water, 1.0 μl NEB Buffer 4, 0.1 μL ExoT (NEB, Ipswich, MA) and 0.25 μL rSAP (NEB, Ipswich, MA) incubated at 37°C for 30 min and at 80°C for 15 min, and stored at -20°C. Sequencing was carried out by Eurofins Genomics (Louisville, KY). Strains expressing xylose-inducible CFP, GFP, YFP, or mCherry from a plasmid integrated at the *xylX* locus. Strains with the Δ*xylB* allele and are incapable of metabolizing the xylose inducer and allow stable expression of the fluorescent protein in rich media.

### Mucoidy assay

We qualitatively assessed mucoidy by patching each strain on high-sugar medium, incubating at 30°C for 48 hours, and storing at 4°C for 24–48 hours before scoring or photographing. Non-mucoid EPS^-^ colonies and patches appear dry and dull, while mucoid EPS^+^ colonies and patches appear moist and shiny when grown under these conditions. Strains producing intermediate amounts of EPS also appear moist and shiny, but to a lesser degree than EPS^+^ strains.

### Synchrony capacity assay

*C*. *crescentus* cells divide asymmetrically to yield a flagellated swarmer cell and a stalked cell; exponentially growing *C*. *crescentus* cultures contain a mixture of these cell types. In EPS^+^ strains, the stalked and predivisional cells can be physically separated from newly born swarmer cells by centrifugation in viscous media. To measure the EPS phenotype of our strains, we quantified the capacity of these strains to be synchronized, liquid cultures were grown to an optical density at 660 nm (OD_660_) of 0.1 to 0.3. Percoll (50 μl; GE Healthcare, Chicago, IL) was added to 500 μl of culture and spun at 13k rpm (Eppendorf 5430R, Hauppauge, NY) for 1 minute. Under these conditions, all cells of strain NA1000ΔMGE collect in the pellet, while only swarmer cells pellet in strain NA1000, leaving the stalked and predivisional cells in a suspension as part of the supernatant. The supernatant and cell suspension were collected, and the pellet was resuspended in 500 μl fresh M2G. We measured the OD_660_ of the supernatant (OD_s_) containing the cell suspension and the OD_660_ of the resuspended cell pellet (OD_p_) separately using a Jenway 6320D spectrophotometer (Staffordshire, UK). To control for differences in total culture density, we defined synchrony capacity as OD_s_/(OD_s_ +OD_p_).

### Lectin binding assay (microscopy)

Binding of Texas Red-*Lotus tetragonolobus* lectin to *Caulobacter* cells was used to assess whether the cells produce EPS. The procedure was previously described in [46] and modified as follows: NA1000 (EPS^+^) and CB15-GFP (EPS^-^) were grown overnight in M2X. Cells were harvested independently (200 μL) by centrifugation (5,000 x g for 3 minutes), resuspended in sterile water, and mixed 50:50 (100 μL each strain). Texas Red conjugated *Lotus tetragonolobus* lectin was added to a final concentration of 0.1 mg/mL (EY Laboratories, San Mateo, CA), incubated at room temperature for 2 hours, and washed three times in water (1 mL). The final pellet was resuspended in water (20 μL) and 1–2 μL was imaged on an agarose pad (1% agarose in M2X) with a Zeiss LSM710 (Oberkochen, Germany).

### Dextran exclusion assay

Methods for visualization of EPS were modified from [24,36]. Briefly, visualization and quantification of the EPS zones of exclusion was carried out as follows: *Caulobacter* strains of different backgrounds (NA1000Δ*xylB*, NA1000Δ*xylB*ΔMGE, NA1000Δ*xylB*Δ*gmd1*, or NA1000Δ*xylB*Δ*gmd2*) expressing xylose-inducible GFP, YFP, or CFP from a plasmid (pXGFPC-2, pXYFPC-2 or pXCFPC-2, respectively, Table 1) were grown independently in M2X supplemented with 3% sucrose to an OD600 = 0.6 (±0.05) prior to the start of the procedure, harvested (500 μL) via centrifugation at 14,000 x g for 5 minutes in an Eppendorf Minispin Plus (Hauppauge, NY), and resuspended with 30μl of 0.5X PBS. Concentrated bacterial suspensions were combined and mixed (10 μL) mixed with TRITC-dextran (5μL, 2,000,000 MW, 10mg/ml, ThermoFisher Scientific, Waltham, MA) and ProLong Live Antifade Reagent (1 μL, ThermoFisher Scientific, Waltham, MA). Samples (2 μL) were transferred onto clean glass slides and cover-slipped. Pressure was applied to the coverslip to ensure equal distribution and fit of TRITC-dextran around cells. Beeswax was used seal the sample and affix the coverslip in place. Samples were imaged on a Zeiss LSM710 (Oberkochen, Germany) and zones of exclusion were measured using Zen Blue software (Carl Zeiss, Oberkochen, Germany). Only isolated cells that could be unambiguously identified as expressing a particular fluorescent protein (GFP, YFP, or CFP) were included in the final data set (Fig 2C). Zones of exclusion were normalized to the average NA1000 zone of exclusion for each experiment (i.e. strains that were mixed and visualized together). Relative zones of exclusion were compared between strains using ANOVA with Bonferroni correction for multiple comparisons in Prism 6.0h (GraphPad Software, Inc., La Jolla, CA).

### Phage adsorption assay

To establish the role of EPS capsulation on bacteriophage adsorption, we exposed mucoid EPS^+^ (NA1000) and non-mucoid EPS^-^ (NA1000ΔMGE) *C*. *crescentus* strains to phage CR30 [25]. Samples were collected periodically and unadsorbed phage remaining in the lysate were titered on a susceptible, EPS^-^ host strain (NA1000ΔMGE). Bacterial strains were grown overnight at 30°C in 5 mL liquid PYE, diluted to an OD600 of 0.2 (Ultrospec 10 Cell Density Meter, GE Healthcare Life Sciences, Pittsburgh, PA), and 3 mL dilute culture was inoculated with 200 μL of 10^4^ PFU/mL phage CR30 lysate and vortexed. Samples (500 μL at 0, 20, and 40 minutes) were transferred to microcentrifuge tubes containing 100 μL chloroform and immediately vortexed. Cell debris and chloroform were separated from the phage lysate by centrifugation for 5 min at 9.7k x g (MiniSpin Plus, Eppendorf, Hauppauge, NY). Lysates were titered by mixing 200 μL host strain (NA1000ΔMGE, OD6_00_ = 0.4) with 20 μL lysate. After a 30-minute incubation (room temperature) 3 mL of warm (45°C) PYE-glucose top agar was added poured onto 60-mm PYE-glucose plates. Cooled top agar lawns were incubated overnight at 30°C before plaques were counted.

### Phage survival assays

#### Endpoint phage survival assay

Phage susceptibility of *C*. *crescentus* was determined by examining growth after inoculation with phage CR30. Liquid cultures were grown overnight in M_15_HIGG media [45] to an OD_660_ of at least 0.15, diluted to an OD_660_ of 0.05 (in duplicate), allowed to grow for 20 minutes and one of the cultures was inoculated with phage CR30 to a final concentration of 2.7x10^6^ pfu/ml, the other was retained as an uninoculated control. After 18 hours of growth, percent survival was calculated as (OD_660_ phage inoculated culture) / (OD_660_ uninoculated control culture) x 100 and survival was compared using ANOVA with Bonferroni correction for multiple comparisons using Prism 6.0h (GraphPad Software, Inc., La Jolla, CA).

#### High-throughput phage survival assay (timecourse)

Population survival in the presence of phage CR30 were measured in 750 μL cultures in PYE at 30°C with shaking (244.5 rpm, 2.5 mm radius) in an Infinite Pro M1000 microplate reader (Tecan, Männedorf, Switzerland). Fresh overnight cultures were diluted to an OD_660_ of 0.03 (Ultrospec 10 Cell Density Meter, GE Healthcare Life Sciences, Pittsburgh, PA) in fresh PYE, distributed into a 48-well flat bottom plate (Genesee Scientific, San Diego, CA), and outgrown for 2 hours in an Infinite Pro M1000 microplate reader (Tecan, Männedorf, Switzerland). Phage lysate was added to a final concentration of 2.6x10^6^ pfu/mL with a predicted multiplicity of infection (MOI) of 0.005–0.01 and the plate was returned to the reader. Throughout the duration of the experiment, optical density measurements were taken every 15 min at an OD_660_ for 15 hours.

### Measurement of population doubling time

Doubling times were measured in 750 μL cultures in PYE at 30°C with shaking (244.5 rpm, 2.5 mm radius) in an Infinite Pro M1000 microplate reader (Tecan, Männedorf, Switzerland). Optical density measurements were taken every 15 min at an OD_660_ for 6–10 hours and fit to a single exponential-growth function in Prism 6.0h (GraphPad Software, Inc., La Jolla, CA). Quantitative differences in growth rates were compared between strains (NA1000 and NA1000ΔMGE) using two-tailed t-test in Prism 6.0h (GraphPad Software, Inc., La Jolla, CA).

### Competition experiments

To measure pairwise competitive ability between our strains, we used strains expressing xylose-inducible GFP or mCherry from a plasmid (pXGFPC-2 or pXCHYC-2, respectively) integrated at the *xylX* locus in a Δ*xylB* background that allows stable expression of the fluorescent protein in rich media (Table 1). All cultures in this experiment were grown in PYE supplemented with 50 μg/mL Kanyamycin and 0.01% xylose. Fluorescence and OD_660_ were monitored in an Infinite Pro M1000 microplate reader (Tecan, Männedorf, Switzerland) using the following excitation, emission, gain, and z-position settings, respectively: mCherry 580 nm, 625 nm, 179, 20185; GFP 483 nm, 535 nm, 135, 19857. Fresh overnight cultures were added to a non-treated 48-well plate (750 μL per well, Genesee Scientific, San Diego, CA). Pure cultures of each fluorescent strain were included on each plate as reference controls for overall fluorescent protein signal. All competitions were prepared by mixing equal volumes (375 μL) of OD-matched starting cultures expressing different fluorescent proteins (one GFP strain and one mCherry strain). Fluorescence and OD_660_ measurements were made upon initial mixing and after each 24-hour incubation period, prior to culture dilution (10 μL overnight culture into 740 μL fresh PYE-Kan-Xyl) and subsequent incubation. Plates were incubated at 30°C with shaking (280 RPM) in an Infors Minitron (Bottmingen, Switzerland). The read-dilute-incubate procedure was repeated daily for 8 days. Raw fluorescence measurements were normalized against the average fluorescence signal from pure cultures of the matched strain and timepoint (n = 8) as well as the average fluorescence signal at day 1. Data were analyzed at day 1 and 8 of the experiment using ANOVA with Bonferroni correction for multiple comparisons in Prism 6.0h (GraphPad Software, Inc., La Jolla, CA)

## Supporting information

S1 Dataset EPS, phage CR30 susceptibility, and competition datasets. Tab 1: Fig 2D Zones of Exlusion contains the data for quantification of EPS (relative zones of TRITC-dextran) for NA1000, NA1000ΔMGE, NA1000Δ*gmd2*, and NA1000Δ*gmd1* (Fig 2D). Relative zones of exclusion were calculated by normalizing to the average NA1000 zone of exclusion for each experiment (i.e. strains that were mixed and visualized together). Tab 2: Fig 3A Phage Adsorption contains quantified unadsorbed phage CR30 (pfu/mL) for the phage adsorption timecourse presented in Fig 3A. Tab 3: Fig 3B Phage Percent Adsorption contains data for the 40 minute endpoint phage adsorption data (percent adsorbed relative to time 0) presented in Fig 3B. Tab 4: Fig 3C Phage Percent Survival contains data for the endpoint phage CR30 survival experiment presented in Fig 3C. Percent survival was calculated relative to an uninoculated control. Tab 5: Fig 3D Phage Timecourse contains data presented in Fig 3D showing the detailed phage CR30 effects on culture growth and lysis during a 15 hour incubation. Tab 6: Doubling Times contains the NA1000 and NA1000ΔMGE culture doubling times in PYE media (data reported in text). Tab 7: Fig 5 Competition contains normalized fluorescence data for all configurations of the competition experiment presented in Fig 5. Fluorescence data were first normalized to the average fluorescence of a pure culture of the appropriate strain and then to the average fluorescence on day 1 of the experiment. Tab 8: S1 Fig Synchrony Capacity contains calculated synchrony capacity data used to assess the presence of EPS in NA1000, NA1000ΔMGE, NA1000Δ469, NA1000Δ*fcl*, and NA1000Δ*gmd2*. Tab 9: S2 Fig Raw Fluorescence contains florescence measurements from pure cultures of GFP or mCherry expressing strains used in the competition experiments.
(XLSX)

S1 Fig Assembly of EPS in *C*. *crescentus* requires functional GDP-fucose synthase (*fcl*) and glycosyltransferase (CCNA_00469). (A) EPS phenotypes on PYE-sucrose. NA1000 has a fully mucoid (EPS^+^) phenotype and NA1000ΔMGE has a dry, non-mucoid (EPS^-^) phenotype when cultured on PYE-sucrose. NA1000Δ*gmd2* shows reduced EPS expression (also see Fig 2). Both NA1000Δ*fcl* and NA1000ΔCCNA_00469 express a dry, non-mucoid (EPS^-^) phenotype. (B) Quantitative measures of synchrony capacity as a proxy for cell buoyancy and EPS production [16,24]. Strains that produce EPS (NA1000 (black bar), NA1000Δ*gmd2* (orange bar) are synchronizable while those lacking EPS (NA1000ΔMGE (blue bar), NA1000Δ*fcl* (yellow bar), NA1000ΔCCNA_00469 (white bar) are not and are indicated with asterisks (*) (ANOVA F(4, 77) = 33.44 p < 0.0001; NA1000 vs. NA1000ΔMGE t(77) = 8.960 p< 0.0001, NA1000 vs. NA1000Δ*469* t(77) = 7.690 p< 0.0001, NA1000 vs. NA1000Δ*fcl* t(77) = 7.119 p< 0.0001, NA1000 vs. NA1000Δ*gmd2* t(77) = 1.039 p> 0.9999, NA1000ΔMGE vs. NA1000Δ*469* t(77) = 0.1513 p> 0.9999, NA1000ΔMGE vs. NA1000Δ*fcl* t(77) = 0.8848 p> 0.9999, NA1000ΔMGE vs. NA1000Δ*gmd2* t(77) = 6.298 p< 0.0001, NA1000Δ*469* vs. NA1000Δ*fcl* t(77) = 0.6675 p> 0.9999, NA1000Δ*469* vs. NA1000Δ*gmd2* t(77) = 5.620 p< 0.0001, NA1000Δ*fcl* vs. NA1000Δ*gmd2* t(77) = 5.064 p< 0.0001).
(TIF)

S2 Fig Raw fluorescence measurements from six strains used in competition experiments. Average raw fluorescence measured in arbitrary units (AU) of pure cultures of the NA1000-GFP (n = 288), NA1000ΔMGE-GFP (n = 279), NA1000Δ*gmd2*-GFP (n = 288), NA1000-mCherry (n = 288), NA1000ΔMGE-mCherry (n = 250), and NA1000Δ*gmd2*-mCherry (n = 288) show that each strain has a characteristic, reproducible brightness that is independent of its EPS phenotype. Notably, NA1000-GFP and NA1000ΔMGE-GFP are not significantly different nor are NA1000ΔMGE and NA1000Δ*gmd2* (ANOVA F(5, 1675) = 52.90 p<0.001; NA1000-GFP vs. NA1000ΔMGE-GFP t(1675) = 1.004 p> 0.9999, NA1000-GFP vs. NA1000Δ*gmd2-*GFP t(1675) = 8.373 p< 0.0001, NA1000ΔMGE-GFP vs. NA1000Δ*gmd2*-GFP t(1675) = 9.310 p> 0.0001, NA1000-mCherry vs. NA1000ΔMGE-mCherry t(1675) = 7.036 p< 0.0001, NA1000-mCherry vs. NA1000Δ*gmd2-*mCherry t(1675) = 8.856 p< 0.0001, NA1000ΔMGE-mCherry vs. NA1000Δ*gmd2*-mCherry t(1675) = 1.502 p< 0.9999).
(TIF)

S3 Fig Disadvantageous effect of EPS production in phage-free environments is unlikely to be the result of new mutation. Individual trials of competition experiments (Fig 5) in which strains expressing different fluorescent proteins (GFP, mCherry) were mixed and relative fluorescence measured over the course of 8 days. (A, B) Control experiments where strains of the same genetic background expressing different fluorescent proteins were mixed. (C, D) Experiments where NA1000 and NA1000ΔMGE were mixed. (E, F) Experiments where NA1000 and NA1000Δ*gmd2* were mixed. (G, H) Experiments where NA1000ΔMGE and NA1000Δ*gmd2* were mixed. Panels on the left (A, C, E, G) show the data color-coded by fluorescent protein (GFP, green; mCherry, red). Panels on the right show the data color-coded by strain (NA1000, black; NA1000ΔMGE, blue; NA1000Δ*gmd2*, orange). In a single trial (NA1000 vs. NA1000ΔMGE), rapid divergence between the two strains is consistent with acquisition of an advantageous mutation followed by rapid selection and fixation (see panels C and D, bold, dashed lines marked with arrow heads). In all other cases (n = 284 mixed cultures) the divergence between strains does not occur (control experiments) or occurs slowly over the course of the experiment.
(TIF)

## References

[1] Tytgat HLP, Lebeer S. The sweet tooth of bacteria: common themes in bacterial glycoconjugates. Microbiol Mol Biol Rev. 2014;78: 372–417. doi: 10.1128/MMBR.00007-14 25184559 10.1128/MMBR.00007-14 PMC4187687

[2] Wilkinson JF. The extracellualr polysaccharides of bacteria. Bacteriol Rev. American Society for Microbiology; 1958;22: 46–73. 10.1128/br.22.1.46-73.1958 PMC180929 13522509

[3] Ferenci T. Trade-off Mechanisms Shaping the Diversity of Bacteria. Trends Microbiol. 2016;24: 209–223. doi: 10.1016/j.tim.2015.11.009 26705697 10.1016/j.tim.2015.11.009

[4] Nadell CD, Bassler BL. A fitness trade-off between local competition and dispersal in *Vibrio cholerae* biofilms. Proc Natl Acad Sci USA. 2011;108: 14181–14185. doi: 10.1073/pnas.1111147108 21825170 10.1073/pnas.1111147108 PMC3161532

[5] Ghoul M, Mitri S. The Ecology and Evolution of Microbial Competition. Trends Microbiol. 2016;24: 833–845. doi: 10.1016/j.tim.2016.06.011 27546832 10.1016/j.tim.2016.06.011

[6] Hibbing ME, Fuqua C, Parsek MR, Peterson SB. Bacterial competition: surviving and thriving in the microbial jungle. Nat Rev Microbiol. 2010;8: 15–25. doi: 10.1038/nrmicro2259 19946288 10.1038/nrmicro2259 PMC2879262

[7] Behrends V, Ryall B, Zlosnik JEA, Speert DP, Bundy JG, Williams HD. Metabolic adaptations of *Pseudomonas aeruginosa* during cystic fibrosis chronic lung infections. Environ Microbiol. 2013;15: 398–408. doi: 10.1111/j.1462-2920.2012.02840.x 22882524 10.1111/j.1462-2920.2012.02840.x

[8] Rodriguez-Valera F, Martin-Cuadrado A-B, Rodriguez-Brito B, Pašić L, Thingstad TF, Rohwer F, et al Explaining microbial population genomics through phage predation. Nat Rev Microbiol. 2009;7: 828–836. doi: 10.1038/nrmicro2235 19834481 10.1038/nrmicro2235

[9] Wasik BR, Turner PE. On the biological success of viruses. Annu Rev Microbiol. 2013;67: 519–541. doi: 10.1146/annurev-micro-090110-102833 23808330 10.1146/annurev-micro-090110-102833

[10] Labrie SJ, Samson JE, Moineau S. Bacteriophage resistance mechanisms. Nat Rev Microbiol. 2010;8: 317–327. doi: 10.1038/nrmicro2315 20348932 10.1038/nrmicro2315

[11] Díaz-Muñoz SL, Koskella B. Bacteria-phage interactions in natural environments. Adv Appl Microbiol. 2014;89: 135–183. doi: 10.1016/B978-0-12-800259-9.00004-4 25131402 10.1016/B978-0-12-800259-9.00004-4

[12] Scanlan PD, Buckling A. Co-evolution with lytic phage selects for the mucoid phenotype of *Pseudomonas fluorescens* SBW25. ISME J. 2011;6: 1148–1158. doi: 10.1038/ismej.2011.174 22189495 10.1038/ismej.2011.174 PMC3358020

[13] Peyraud R, Cottret L, Marmiesse L, Gouzy J, Genin S. A resource allocation trade-off between virulence and proliferation drives metabolic versatility in the plant pathogen *Ralstonia solanacearum*. PLoS Pathog. 2016;12: e1005939 doi: 10.1371/journal.ppat.1005939 27732672 10.1371/journal.ppat.1005939 PMC5061431

[14] Madsen JS, Lin Y-C, Squyres GR, Price-Whelan A, de Santiago Torio A, Song A, et al Facultative control of matrix production optimizes competitive fitness in *Pseudomonas aeruginosa* PA14 biofilm models. Kivisaar M, editor. Appl Environ Microbiol. 2015;81: 8414–8426. doi: 10.1128/AEM.02628-15 26431965 10.1128/AEM.02628-15 PMC4644639

[15] van Gestel J, Weissing FJ, Kuipers OP, Kovács AT. Density of founder cells affects spatial pattern formation and cooperation in *Bacillus subtilis* biofilms. ISME J. 2014;8: 2069–2079. doi: 10.1038/ismej.2014.52 24694715 10.1038/ismej.2014.52 PMC4184017

[16] Marks ME, Castro-Rojas CM, Teiling C, Du L, Kapatral V, Walunas TL, et al The genetic basis of laboratory adaptation in *Caulobacter crescentus*. J Bacteriol. 2010;192: 3678–3688. doi: 10.1128/JB.00255-10 20472802 10.1128/JB.00255-10 PMC2897358

[17] Leiser OP, Merkley ED, Clowers BH, Deatherage Kaiser BL, Lin A, Hutchison JR, et al Investigation of *Yersinia pestis* laboratory adaptation through a combined genomics and proteomics approach. Robinson DA, editor. PLoS ONE. 2015;10: e0142997 doi: 10.1371/journal.pone.0142997 26599979 10.1371/journal.pone.0142997 PMC4658026

[18] Sterken MG, Snoek LB, Kammenga JE, Andersen EC. The laboratory domestication of *Caenorhabditis elegans*. Trends Genet. 2015;31: 224–231. doi: 10.1016/j.tig.2015.02.009 25804345 10.1016/j.tig.2015.02.009 PMC4417040

[19] Gompel N, Prud'homme B. The causes of repeated genetic evolution. Dev Biol. 2009;332: 36–47. doi: 10.1016/j.ydbio.2009.04.040 19433086 10.1016/j.ydbio.2009.04.040

[20] Pekkonen M, Ketola T, Laakso JT. Resource availability and competition shape the evolution of survival and growth ability in a bacterial community. PLoS ONE. 2013;8: e76471 doi: 10.1371/journal.pone.0076471 24098791 10.1371/journal.pone.0076471 PMC3787024

[21] Nierman WC, Feldblyum TV, Laub MT, Paulsen IT, Nelson KE, Eisen JA, et al Complete genome sequence of *Caulobacter crescentus*. PNAS. 2001;98: 4136–4141. doi: 10.1073/pnas.061029298 11259647 10.1073/pnas.061029298 PMC31192

[22] Evinger M, Agabian N. Envelope-associated nucleoid from *Caulobacter crescentus* stalked and swarmer cells. J Bacteriol. 1977;132: 294–301. 334726 10.1128/jb.132.1.294-301.1977 PMC221855

[23] Ravenscroft N, Walker SG, Dutton GG, Smit J. Identification, isolation, and structural studies of extracellular polysaccharides produced by *Caulobacter crescentus*. J Bacteriol. 1991;173: 5677–5684. 1885545 10.1128/jb.173.18.5677-5684.1991 PMC208297

[24] Ardissone S, Fumeaux C, Bergé M, Beaussart A, Théraulaz L, Radhakrishnan SK, et al Cell cycle constraints on capsulation and bacteriophage susceptibility. Elife. 2014;3: 1695 doi: 10.7554/eLife.03587 25421297 10.7554/eLife.03587 PMC4241560

[25] Ely B, Johnson RC. Generalized Transduction in *Caulobacter crescentus*. Genetics. 1977;87: 391–399. 17248770 10.1093/genetics/87.3.391 PMC1213749

[26] Abel S, Bucher T, Nicollier M, Hug I, Kaever V, Abel Zur Wiesch P, et al Bi-modal distribution of the second messenger c-di-GMP controls cell fate and asymmetry during the *Caulobacter* cell cycle. PLoS Genet. 2013;9: e1003744 doi: 10.1371/journal.pgen.1003744 24039597 10.1371/journal.pgen.1003744 PMC3764195

[27] Chang A, Schomburg I, Placzek S, Jeske L, Ulbrich M, Xiao M, et al BRENDA in 2015: exciting developments in its 25th year of existence. Nucleic Acids Res. 2015;43: D439–46. doi: 10.1093/nar/gku1068 25378310 10.1093/nar/gku1068 PMC4383907

[28] Caspi R, Billington R, Ferrer L, Foerster H, Fulcher CA, Keseler IM, et al The MetaCyc database of metabolic pathways and enzymes and the BioCyc collection of pathway/genome databases. Nucleic Acids Res. 2016;44: D471–80. doi: 10.1093/nar/gkv1164 26527732 10.1093/nar/gkv1164 PMC4702838

[29] Schrader JM, Zhou B, Li G-W, Lasker K, Childers WS, Williams B, et al The coding and noncoding architecture of the *Caulobacter crescentus* genome. PLoS Genet. 2014;10: e1004463 doi: 10.1371/journal.pgen.1004463 25078267 10.1371/journal.pgen.1004463 PMC4117421

[30] Jones MD, Vinogradov E, Nomellini JF, Smit J. The core and O-polysaccharide structure of the *Caulobacter crescentus* lipopolysaccharide. Carbohydr Res. 2015;402: 111–117. doi: 10.1016/j.carres.2014.10.003 25498010 10.1016/j.carres.2014.10.003

[31] Cabeen MT, Murolo MA, Briegel A, Bui NK, Vollmer W, Ausmees N, et al Mutations in the Lipopolysaccharide biosynthesis pathway interfere with crescentin-mediated cell curvature in *Caulobacter crescentus*. J Bacteriol. 2010;192: 3368–3378. doi: 10.1128/JB.01371-09 20435724 10.1128/JB.01371-09 PMC2897673

[32] Awram P, Smit J. Identification of lipopolysaccharide O antigen synthesis genes required for attachment of the S-layer of *Caulobacter crescentus*. Microbiology (Reading, Engl). 2001;147: 1451–1460. doi: 10.1099/00221287-147-6-1451 11390676 10.1099/00221287-147-6-1451

[33] Toporowski MC, Nomellini JF, Awram P, Levi A, Smit J. Transcriptional regulation of the S-layer protein type I secretion system in *Caulobacter crescentus*. FEMS Microbiol Lett. 2005;251: 29–36. doi: 10.1016/j.femsle.2005.07.028 16111836 10.1016/j.femsle.2005.07.028

[34] Ried JL, Collmer A. An nptI-sacB-sacR cartridge for constructing directed, unmarked mutations in gram-negative bacteria by marker exchange-eviction mutagenesis. Gene. 1987;57: 239–246. doi: 10.1016/0378-1119(87)90127-2 3319780 10.1016/0378-1119(87)90127-2

[35] Thanbichler M, Iniesta AA, Shapiro L. A comprehensive set of plasmids for vanillate- and xylose-inducible gene expression in *Caulobacter crescentus*. Nucleic Acids Res. 2007;35: e137–e137. doi: 10.1093/nar/gkm818 17959646 10.1093/nar/gkm818 PMC2175322

[36] Gates MA, Thorkildson P, Kozel TR. Molecular architecture of the *Cryptococcus neoformans* capsule. Mol Microbiol. 2004;52: 13–24. doi: 10.1111/j.1365-2958.2003.03957.x 15049807 10.1111/j.1365-2958.2003.03957.x

[37] Edwards P, Smit J. A transducing bacteriophage for *Caulobacter crescentus* uses the paracrystalline surface layer protein as a receptor. J Bacteriol. 1991;173: 5568–5572. doi: 10.1128/jb.173.17.5568–5572.1991 1885534 10.1128/jb.173.17.5568-5572.1991 PMC208274

[38] Boutte CC, Crosson S. Bacterial lifestyle shapes stringent response activation. Trends Microbiol. 2013;21: 174–180. doi: 10.1016/j.tim.2013.01.002 23419217 10.1016/j.tim.2013.01.002 PMC4238387

[39] Stephens C, Christen B, Fuchs T, Sundaram V, Watanabe K, Jenal U. Genetic analysis of a novel pathway for D-xylose metabolism in *Caulobacter crescentus*. J Bacteriol. 2007;189: 2181–2185. doi: 10.1128/JB.01438-06 17172333 10.1128/JB.01438-06 PMC1855722

[40] Stephens C, Christen B, Watanabe K, Fuchs T, Jenal U. Regulation of D-Xylose Metabolism in *Caulobacter crescentus* by a LacI-Type Repressor. J Bacteriol. 2007;189: 8828–8834. doi: 10.1128/JB.01342-07 17933895 10.1128/JB.01342-07 PMC2168598

[41] Samson JE, Magadán AH, Sabri M, Moineau S. Revenge of the phages: defeating bacterial defences. Nat Rev Microbiol. 2013;11: 675–687. doi: 10.1038/nrmicro3096 23979432 10.1038/nrmicro3096

[42] Juhas M, van der Meer JR, Gaillard M, Harding RM, Hood DW, Crook DW. Genomic islands: tools of bacterial horizontal gene transfer and evolution. Fems Microbiol Rev. 2009;33: 376–393. doi: 10.1111/j.1574-6976.2008.00136.x 19178566 10.1111/j.1574-6976.2008.00136.x PMC2704930

[43] Boyd EF, Almagro-Moreno S, Parent MA. Genomic islands are dynamic, ancient integrative elements in bacterial evolution. Trends Microbiol. 2009;17: 47–53. doi: 10.1016/j.tim.2008.11.003 19162481 10.1016/j.tim.2008.11.003

[44] Ely B. Genetics of *Caulobacter crescentus*. Meth Enzymol. 1991;204: 372–384. doi: 10.1016/0076-6879(91)04019-K 1658564 10.1016/0076-6879(91)04019-k

[45] Smit J, Hermodson M, Agabian N. *Caulobacter crescentus* pilin. Purification, chemical characterization, and NH2-terminal amino acid sequence of a structural protein regulated during development. J Biol Chem. 1981;256: 3092–3097. 7009605

[46] Ma L, Lu H, Sprinkle A, Parsek MR, Wozniak DJ. *Pseudomonas aeruginosa* Psl is a galactose- and mannose-rich exopolysaccharide. J Bacteriol. 2007;189: 8353–8356. doi: 10.1128/JB.00620-07 17631634 10.1128/JB.00620-07 PMC2168683

