# Supplementary figures and images for: Exopolysaccharide production in Caulobacter crescentus: A resource allocation trade-off between protection and proliferation
Source: PLoS One. 2018 Jan 2;13(1):e0190371. doi: 10.1371/journal.pone.0190371 (PMC5749776; doi:10.1371/journal.pone.0190371)

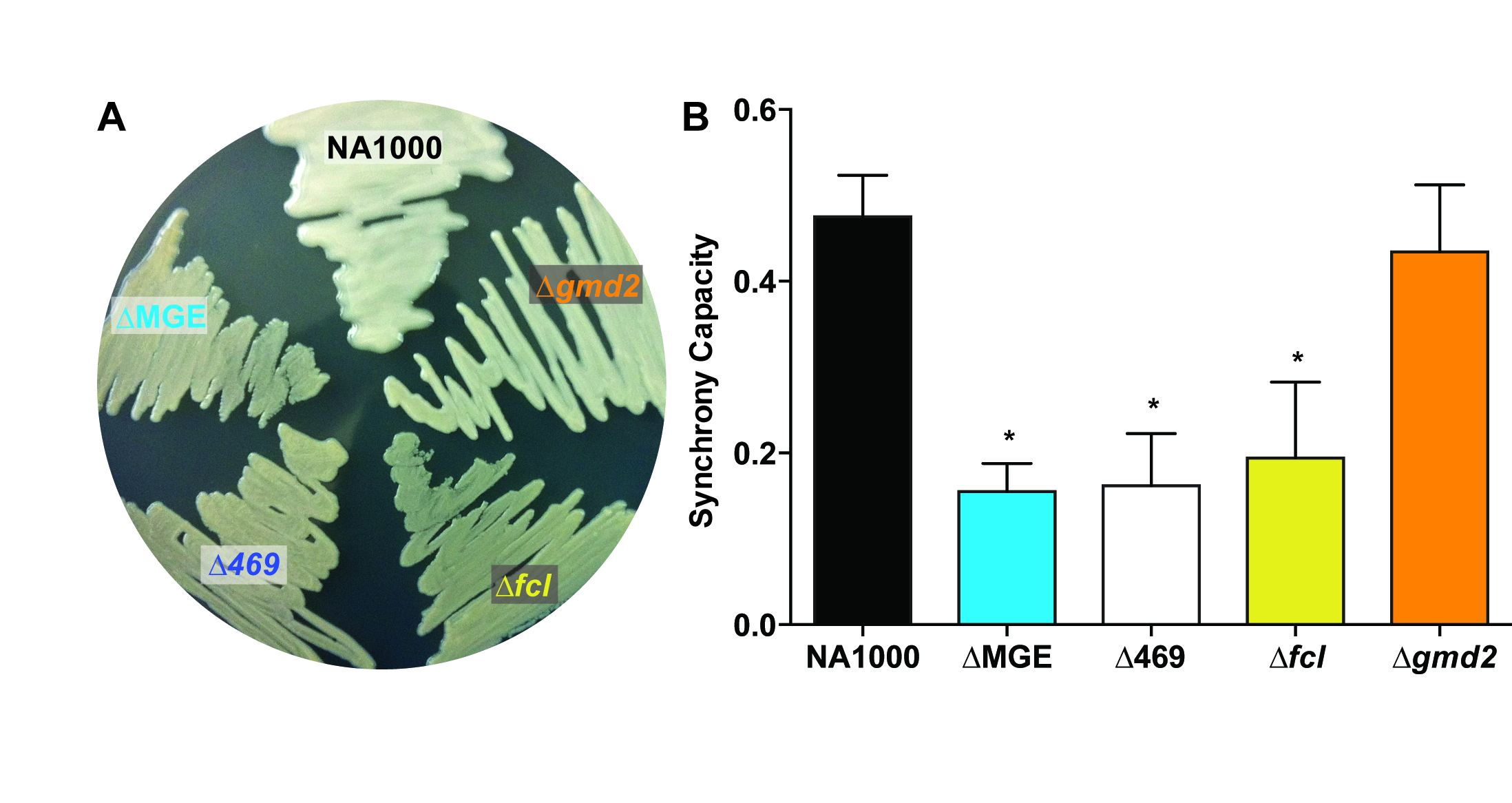

Supplement: S1 Fig — (A) EPS phenotypes on PYE-sucrose. NA1000 has a fully mucoid (EPS+) phenotype and NA1000ΔMGE has a dry, non-mucoid (EPS-) phenotype when cultured on PYE-sucrose. NA1000Δgmd2 shows reduced EPS expression (also see Fig 2). Both NA1000Δfcl and NA1000ΔCCNA_00469 express a dry, non-mucoid (EPS-) phenotype. (B) Quantitative measures of synchrony capacity as a proxy for cell buoyancy and EPS production [16,24]. Strains that produce EPS (NA1000 (black bar), NA1000Δgmd2 (orange bar) are synchronizable while those lacking EPS (NA1000ΔMGE (blue bar), NA1000Δfcl (yellow bar), NA1000ΔCCNA_00469 (white bar) are not and are indicated with asterisks (*) (ANOVA F(4, 77) = 33.44 p < 0.0001; NA1000 vs. NA1000ΔMGE t(77) = 8.960 p< 0.0001, NA1000 vs. NA1000Δ469 t(77) = 7.690 p< 0.0001, NA1000 vs. NA1000Δfcl t(77) = 7.119 p< 0.0001, NA1000 vs. NA1000Δgmd2 t(77) = 1.039 p> 0.9999, NA1000ΔMGE vs. NA1000Δ469 t(77) = 0.1513 p> 0.9999, NA1000ΔMGE vs. NA1000Δfcl t(77) = 0.8848 p> 0.9999, NA1000ΔMGE vs. NA1000Δgmd2 t(77) = 6.298 p< 0.0001, NA1000Δ469 vs. NA1000Δfcl t(77) = 0.6675 p> 0.9999, NA1000Δ469 vs. NA1000Δgmd2 t(77) = 5.620 p< 0.0001, NA1000Δfcl vs. NA1000Δgmd2 t(77) = 5.064 p< 0.0001). (TIF) [file pone.0190371.s002.tif]

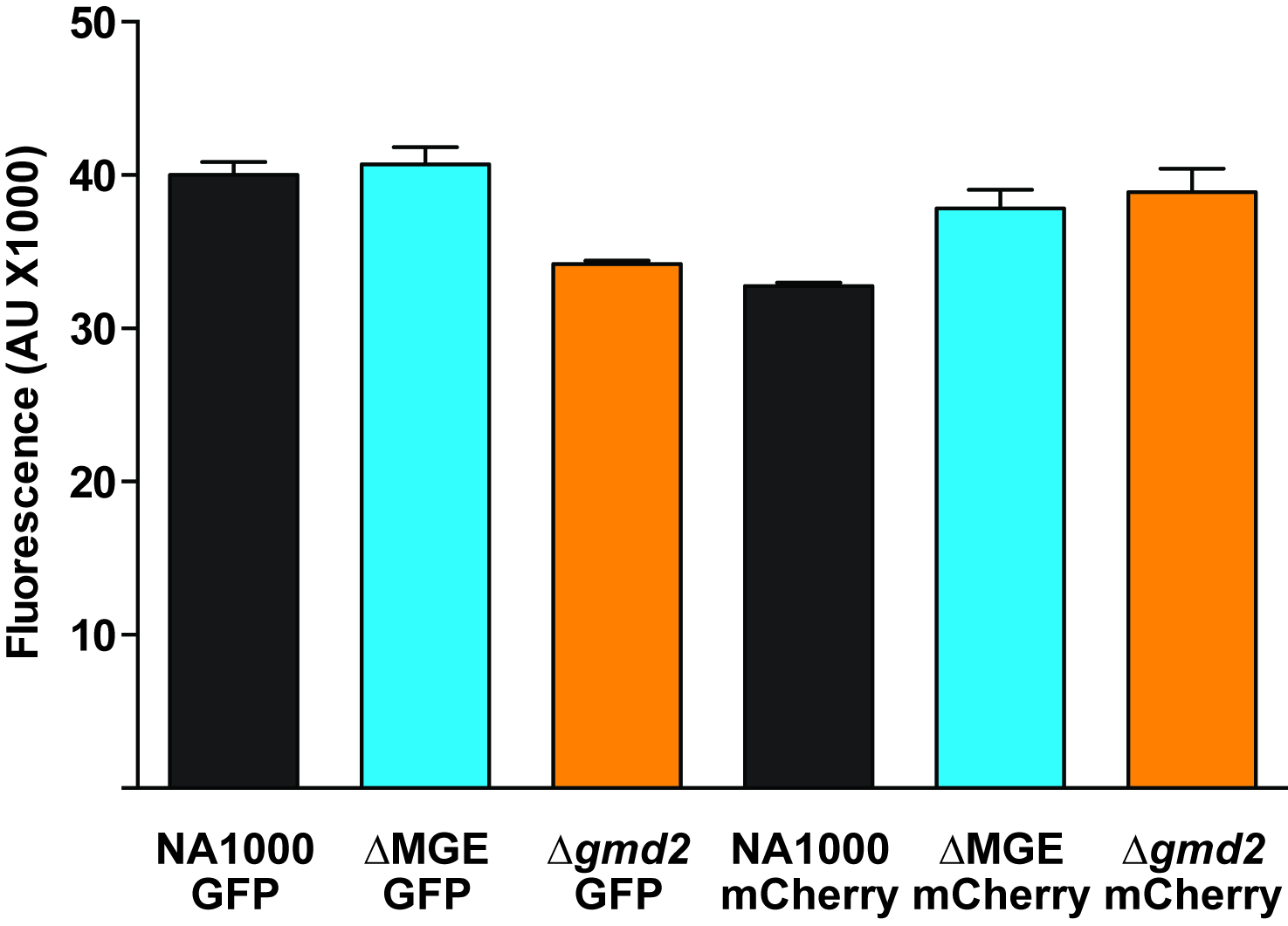

Supplement: S2 Fig — Average raw fluorescence measured in arbitrary units (AU) of pure cultures of the NA1000-GFP (n = 288), NA1000ΔMGE-GFP (n = 279), NA1000Δgmd2-GFP (n = 288), NA1000-mCherry (n = 288), NA1000ΔMGE-mCherry (n = 250), and NA1000Δgmd2-mCherry (n = 288) show that each strain has a characteristic, reproducible brightness that is independent of its EPS phenotype. Notably, NA1000-GFP and NA1000ΔMGE-GFP are not significantly different nor are NA1000ΔMGE and NA1000Δgmd2 (ANOVA F(5, 1675) = 52.90 p<0.001; NA1000-GFP vs. NA1000ΔMGE-GFP t(1675) = 1.004 p> 0.9999, NA1000-GFP vs. NA1000Δgmd2-GFP t(1675) = 8.373 p< 0.0001, NA1000ΔMGE-GFP vs. NA1000Δgmd2-GFP t(1675) = 9.310 p> 0.0001, NA1000-mCherry vs. NA1000ΔMGE-mCherry t(1675) = 7.036 p< 0.0001, NA1000-mCherry vs. NA1000Δgmd2-mCherry t(1675) = 8.856 p< 0.0001, NA1000ΔMGE-mCherry vs. NA1000Δgmd2-mCherry t(1675) = 1.502 p< 0.9999). (TIF) [file pone.0190371.s003.tif]

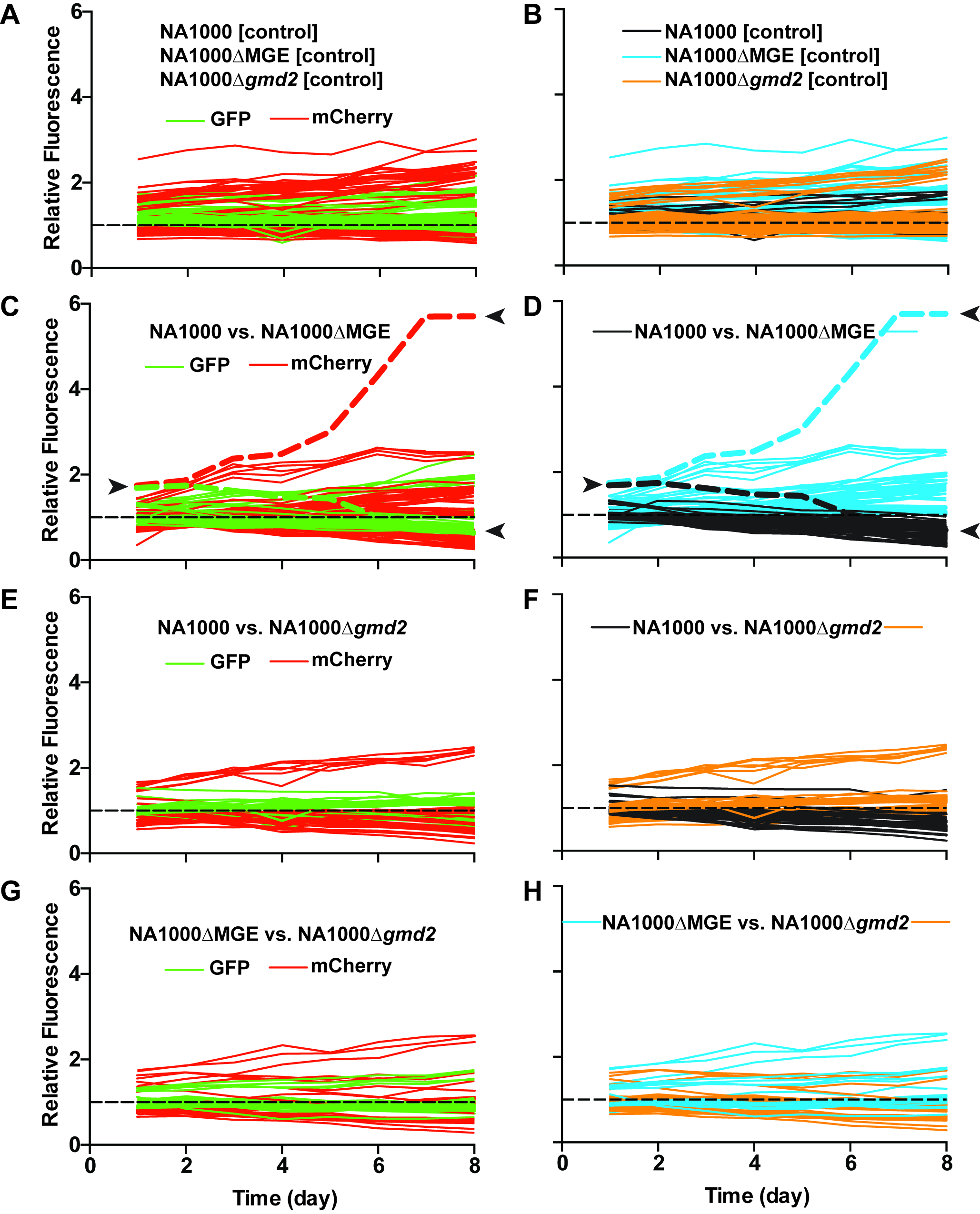

Supplement: S3 Fig — Individual trials of competition experiments (Fig 5) in which strains expressing different fluorescent proteins (GFP, mCherry) were mixed and relative fluorescence measured over the course of 8 days. (A, B) Control experiments where strains of the same genetic background expressing different fluorescent proteins were mixed. (C, D) Experiments where NA1000 and NA1000ΔMGE were mixed. (E, F) Experiments where NA1000 and NA1000Δgmd2 were mixed. (G, H) Experiments where NA1000ΔMGE and NA1000Δgmd2 were mixed. Panels on the left (A, C, E, G) show the data color-coded by fluorescent protein (GFP, green; mCherry, red). Panels on the right show the data color-coded by strain (NA1000, black; NA1000ΔMGE, blue; NA1000Δgmd2, orange). In a single trial (NA1000 vs. NA1000ΔMGE), rapid divergence between the two strains is consistent with acquisition of an advantageous mutation followed by rapid selection and fixation (see panels C and D, bold, dashed lines marked with arrow heads). In all other cases (n = 284 mixed cultures) the divergence between strains does not occur (control experiments) or occurs slowly over the course of the experiment. (TIF) [file pone.0190371.s004.tif]
